# Clinical Applications of Artificial Intelligence—An Updated Overview

**DOI:** 10.3390/jcm11082265

**Published:** 2022-04-18

**Authors:** Ștefan Busnatu, Adelina-Gabriela Niculescu, Alexandra Bolocan, George E. D. Petrescu, Dan Nicolae Păduraru, Iulian Năstasă, Mircea Lupușoru, Marius Geantă, Octavian Andronic, Alexandru Mihai Grumezescu, Henrique Martins

**Affiliations:** 1 “Carol Davila” University of Medicine and Pharmacy, 050474 Bucharest, Romania; stefan.busnatu@umfcd.ro (Ș.B.); alexandra.bolocan@umfcd.ro (A.B.); george.petrescu@umfcd.ro (G.E.D.P.); dan.paduraru@umfcd.ro (D.N.P.); iulian.nastasa@umfcd.ro (I.N.); mircea.lupusoru@umfcd.ro (M.L.); octavian.andronic@umfcd.ro (O.A.); 2 Department of Science and Engineering of Oxide Materials and Nanomaterials, Faculty of Applied Chemistry and Materials Science, Politehnica University of Bucharest, 011061 Bucharest, Romania; adelina.niculescu@upb.ro; 3 Centre for Innovation in Medicine, “Carol Davila” University of Medicine and Pharmacy, 050474 Bucharest, Romania; marius.geanta@kolmedia.ro; 4 Research Institute of the University of Bucharest—ICUB, University of Bucharest, 050657 Bucharest, Romania; 5 Academy of Romanian Scientists, Ilfov No. 3, 50044 Bucharest, Romania; 6 Faculty of Health Sciences, Universidade da Beira Interior, 6200-506 Covilha, Portugal; henrique@henriquemartins.eu

**Keywords:** artificial intelligence, machine learning, deep learning, clinical applications, precision medicine, personalized medicine

## Abstract

Artificial intelligence has the potential to revolutionize modern society in all its aspects. Encouraged by the variety and vast amount of data that can be gathered from patients (e.g., medical images, text, and electronic health records), researchers have recently increased their interest in developing AI solutions for clinical care. Moreover, a diverse repertoire of methods can be chosen towards creating performant models for use in medical applications, ranging from disease prediction, diagnosis, and prognosis to opting for the most appropriate treatment for an individual patient. In this respect, the present paper aims to review the advancements reported at the convergence of AI and clinical care. Thus, this work presents AI clinical applications in a comprehensive manner, discussing the recent literature studies classified according to medical specialties. In addition, the challenges and limitations hindering AI integration in the clinical setting are further pointed out.

## 1. Introduction

Artificial intelligence (AI) has increasingly become an integral part of our life, having an undeniable impact on today’s society. Owing to the growth of computing power, advances in methods and techniques, and the explosion of data, AI has positioned itself as a supportive technology in many domains, ranging from industry to business and education [1,2,3,4].

Despite not having an official definition, AI is generally recognized as the ability to imitate human cognitive functions using machines. Through an ingenious association of computer science, algorithms, machine learning, and data science, AI can solve tasks with a comparable performance to humans or above their level [3,5,6,7].

**Figure 1:**
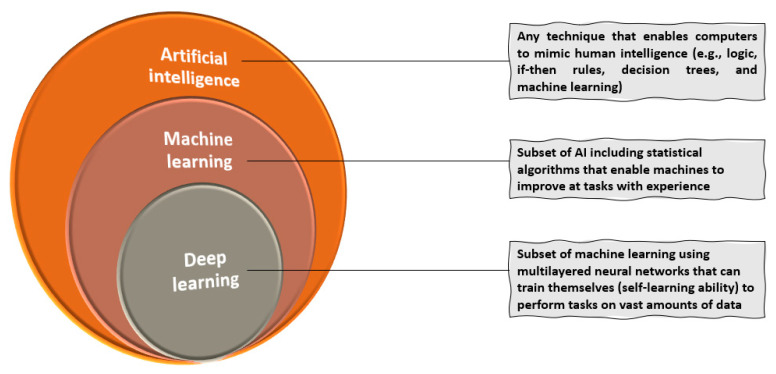
Relationship between AI, ML, and DL. Created based on information from [4,8,9,10].

In more detail, AI comprises any system with the ability to sense, reason, engage, and learn that can be used for various human-like functions, such as understanding digital images, voice recognition, motion, planning, and organization. On the other hand, machine learning (ML) is a subset of AI that uses statistical techniques to endow computer systems with the ability to improve with experience. Specifically, ML encompasses the AI tools that can adapt their models to improve predictions, leading to a progressive performance enhancement for the set task. Theoretically, ML methods can be applied to datasets of any size, yet a larger amount of data provides more experience for training the model. The working principle of ML assumes feeding these features into computational models that can offer insights into the observations, such as clustering similar observations into groups or predicting certain outcomes. Moving further, deep learning (DL) assumes the subclass of ML where algorithms can train themselves due to the “self-learning” ability achieved through a sequential chain of pivotal features from input data. Data representations are automatically mastered by deep neural networks (DNNs) that can learn very complex nonlinear mathematical functions. The term “deep” is used in reference to the number of layers (so-called neurons) or iterations between the input and output. In more detail, input features are fed into the first layer of neurons and propagated towards the output layer, the process being inspired by the information processing principles of biological neurons [1,7,8,9]. The relationship between AI, ML, and DL is visually represented in Figure 1. Moreover, Table 1 briefly defines the most important and relevant concepts concerning AI tools.

In recent years, AI has attracted more and more research interest in medicine, being investigated for a plethora of applications. Numerous studies have evaluated various aspects of the healthcare system, reporting the progress in AI involvement in the prevention, screening, and treatment of diseases and prediction of the prognosis [14,15].

In this context, the present paper thoroughly reviews the most recent potential clinical applications of AI in the fields of cardiology, neurology, oncology, hematology, nephrology, gastroenterology, hepatology, orthopedics, and rheumatology, further focusing on the challenges that limit their introduction to clinical practice. More specifically, this work reviews English language research articles in the fields published in the last 5 years (2018–2022). Studies were retrieved from the Science Direct and Google Scholar databases using combinations between the following keywords: “artificial intelligence”, “machine learning”, “deep learning”, “clinical applications”, “prediction”, “diagnosis”, “screening”, “treatment”, “prognosis”, “cardiology”, “neurology”, “oncology”, “cancer”, “hematology”, “nephrology”, “gastroenterology”, “hepatology”, “orthopedics”, and “rheumatology”. The relevant search results were manually selected; the papers were analyzed and discussed in the main text of the review; and the studies for which at least one of the following performance metrics was available, namely accuracy, precision, sensitivity, specificity, or correlation between the automated and manual measurements, were additionally summarized in a series of tables. The choice of the included medical specialties was based on the available literature data, as other areas were not as explored in recent years or remained at the level of hypothesis/opinion papers.

## 2. Applications of AI in Clinical Care

**Figure 2:**
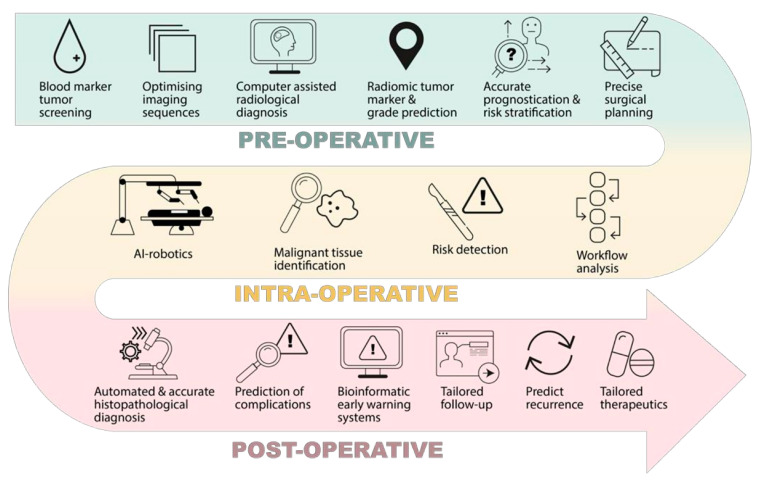
Examples of AI potential applications in clinical care. Reproduced from [6].

Artificial intelligence has faced tremendous advances in recent years, rendering it interesting for applications in a variety of clinical care procedures (Figure 2). In this respect, the following subsections discuss the most recent studies concerning AI involvement in medicine, classified according to medical specialties. Moreover, the studies for which at least one of the following performance metrics was available, namely accuracy, precision, sensitivity, specificity, or correlations between automated and manual measurements, are included in the summative tables.

### 2.1. Cardiology

Cardiovascular diseases represent one of the leading causes of morbidity and mortality worldwide, requiring expensive treatments and posing a burden on both patients and the healthcare system [16]. Introducing AI technology to the field of cardiology holds great promise for improving the prediction and diagnosis of cardiac events and visualizing cardiac anomalies that anticipate patients’ needs and provide personalized medical care. This is particularly appealing in cardiology due to the large amount and variety of available biological data. By properly analyzing and interpreting images, pulse waves, electrocardiograms, and sound information, various algorithms can identify patterns that lead to disease offset or aggravation, helping the cardiologists in choosing the best treatment alternative [15,17].

For instance, Ye et al. [18] developed and validated a model for predicting incident essential hypertension. The authors used a machine learning algorithm for processing electronic health record (EHR) data that generated an ensemble of classification trees and assigned a predictive risk score to each individual. As the model was able to accurately predict incident essential hypertension for the following year, it has been deployed in the state of Maine to provide implications in interventions for hypertension and associated diseases and, subsequentially, improve hypertension care.

Another interesting example was proposed by Tison and colleagues [19], who created a deep neural network for detecting atrial fibrillation using smartwatch data, such as heart rate and step count. The deep neural network (DNN) was trained by the heuristic pretraining method (the network approximated representations of the R–R interval (i.e., the time between heartbeats) without manual labeling of the training data) and validated both against the reference standard 12-lead electrocardiography in a separate cohort of patients undergoing cardioversion and on smartwatch data from ambulatory patients against the reference standard of self-reported persistent AF history. The authors concluded that the combination of smartwatch photoplethysmography and DNN could be a solution for passively detecting AF, with some reduction in sensitivity and specificity against a criterion standard ECG.

In contrast, Picon et al. [20] coupled one-dimensional convolutional neural network (1D-CNN) layers and a long short-term memory (LSTM) network into a deep learning architecture for detecting ventricular fibrillation. The newly proposed DL architecture was compared to 1D-CNN only and to a classical approach based on ventricular fibrillation (VF) detection features and a support vector machine (SVM), outperforming these classifiers. Up to that moment, and according to the authors’ knowledge, this algorithm was the most accurate for VF detection, with the potential to enable an accurate shock or no shock diagnosis in a very short time.

Artificial intelligence was also found to be useful for the classification of aortic stenosis, as demonstrated by Yang et al. [21]. The research group used a feature analysis framework to reduce the features from collected cardio-mechanical signals generated by continuous wavelet transform. Several ML algorithms were compared by performance, while an additional 2D-CNN was developed using CWT coefficients as images. The obtained accuracies validated the effectiveness of the feature selection and classification framework, encouraging the implementation of AI tools in AS classification.

Alternatively, Eberhard et al. [22] evaluated the feasibility of a computed tomography-derived fractional flow reserve (FFR_CT_) in patients presenting to the emergency department with acute chest pain who underwent chest pain-computed tomography (CPCT). As FFR_CT_ allows a noninvasive functional assessment of coronary artery stenosis, involving ML-based software for performing the measurements could only make it more advantageous. By evaluating the agreement between the results from the FFR_CT_ and patient outcomes for a follow-up of three months, the authors concluded that this method was feasible for the tested category of patients. Therefore, this approach can be implemented in clinical care for improving patient triage by reducing the need for further downstream testing. Nonetheless, some limitations were noticed in patients with CT signs of acute plaque rupture, so further research is needed.

Another study conducted by Nguyen et al. [23] proposed an algorithm based on a CNN as a feature extractor and a Boosting classifier for detecting sudden cardiac arrest on an electrocardiogram signal. Their developed shock advice algorithm applied in the automated external defibrillator showed a validated performance, with the accuracy, sensitivity, and specificity above the preexisting algorithms for the same task. Thus, its high and reliable detection represents a potential asset in clinical settings, the correct detection of SCA being essential for improving the survival rate and reducing unnecessary defibrillation.

Retson and colleagues [24] developed a deep learning algorithm for the clinical measurements of the right and left ventricular volumes and functions. MRI images for various clinical indications and pathologies were analyzed with the aid of commercially available software for the automated DL-based and manual contouring of biventricular volumes. Given the promising obtained results, the authors concluded that their algorithm could be used to aid in expert segmentation; however, the model can benefit from expert supervision, especially for solving errors of the basal and apical slices.

Encouraging results were also obtained by Hannun et al. [25], who developed a DNN able to classify 12 rhythm classes based on single-lead electrocardiograms. When validated against an independent test dataset annotated by a consensus committee of board-certified practicing cardiologists, the DNN performance outperformed the specialists, exceeding the average cardiologist sensitivity for all the rhythm classes. Hence, it may be expected that introducing this DL-based approach into the clinical setting could lower the rate of misdiagnosed computerized ECG interpretations and enhance the human ECG interpretation efficiency by properly triaging patients and prioritizing the most urgent conditions.

To summarize the discussion in a clear and easy-to-follow manner, Table 2 comprises the information regarding the objectives of the studies mentioned above, the used AI approaches, the data sources for the developed algorithms, and the performance metrics.

### 2.2. Neurology

The benefits of AI have also attracted attention in the area of clinical neurosciences, as newly developed tools can ensure early detection and improve the management of neurological conditions [26].

Abedi et al. [27] recently investigated ML-based tools for predicting stroke recurrence and identifying the key variables. All the selected algorithms (i.e., logistic regression, XGBoost, gradient boosting machine, random forest, SVM, and decision tree) could be trained to predict the occurrence of long-term ischemic stroke, and laboratory-based variables were highly correlated with stroke recurrence, paving the way for personalized interventions. Another study on acute ischemic stroke was conducted by Rava et al. [28], who researched the matter from the perspective of collateral circulation. AI-based algorithms can accurately and efficiently determine a patient’s degree of collateral flow, being a potential tool for helping in the decision of which patients are eligible for a reperfusion procedure.

Tackling the potential of machine learning as well, Young et al. [29] introduced a ML technique called “Subtype and Stage Interference (SuStaIn)”. SuStaIn can be used for identifying genotypes from imaging alone in neurodegenerative diseases, such as genetic frontotemporal dementia and Alzheimer’s disease. Providing fine-grained patient stratification, SuStaIn can significantly enhance the ability to predict the conversion between diagnostic categories over standard models that ignore the subtype or temporal stage. Thus, it holds great promise in enabling disease subtype discovery and precision medicine. Following similar considerations, Eshaghi and colleagues [30] applied unsupervised ML to brain MRI scans to classify multiple sclerosis based on the pathological features. According to the authors’ findings, the identified subtypes predicted the disability progression and patients’ responses to treatment, SuStaIn being a valuable technique for use in defining groups of patients in interventional studies.

Considerable research interest has been invested in applying AI technology to detecting epilepsy and managing this disease. For instance, Jin et al. [31] coupled machine learning with automated surface-based MRI morphometry, obtaining a robust performance of detecting FCD during presurgical evaluations for patients with pharmacoresistant epilepsy. Using a similar approach, Gleichgerrcht et al. [32] focused on improving the detection of brain abnormalities in temporal lobe epilepsy patients, concluding that ML has the potential to aid in the radiological diagnosis of this disease. Alternatively, Daoud and Bayoumi [33] developed a deep learning-based technique applied to long-term scalp electroencephalogram recordings for predicting incoming epileptic seizures, obtaining the best performance among the state-of-the-art techniques. As recently demonstrated by Quon and colleagues [34], DL also represents a potential solution in automatically classifying intracranial epileptiform discharges (IEDs). Using a template-matching algorithm and a CNN, the authors obtained comparable performances to expert clinical neurophysiologists, confident in their study’s potential practical applications.

Another interesting study for the field of neurology was reported by Qiu et al. [35]. The authors proposed an interpretable DL strategy for the delineation of unique Alzheimer’s disease signatures from multimodal inputs. A fully convolutional network was involved for constructing high-resolution maps of disease probability from the local brain structure to a multilayer perceptron. In this manner, the model could generate precise, intuitive visualization of an individual’s Alzheimer’s disease risk, identifying nuanced neuroimaging signatures for diagnosing this condition.

Research has also been conducted towards improving the diagnosis and care of other neurodegenerative disorders. For example, Shinde et al. [36] established a computer-based analysis technique to create prognostic and diagnostic biomarkers of Parkinson’s disease (PD) by the use of neuromelanin-sensitive MRI (NMS-MRI). NMS-MRI has been involved, as this method can help identify the abnormalities in the substantia nigra pars compacta (SNc) in PD, since condition is characterized by the loss of dopaminergic neurons in the SNc. The proposed CNN-based method offered a testing accuracy superior to contrast ratio-based classification and the radiomics classifier, supporting PD discrimination from atypical parkinsonian syndromes. The authors concluded that their technique might support the radiological diagnosis of PD while facilitating a deeper understanding of the abnormalities in SNc.

Important results were also reported in a study performed on patients in the intensive care unit who had an acute brain injury and were unresponsive to spoken commands. Claassen et al. [37] applied ML to patients’ EEG recordings to detect brain activation in response to commands that patients move their hands. The authors observed in this manner that, early after brain injury, 15% of the clinically unresponsive patients who did not follow spoken motor commands presented EEG evidence of brain activation in response to them.

Artificial intelligence can also be involved in improving brain surgeries. Shahjouei et al. [38] developed an ANN for predicting the safe clipping time (SCT) of temporary artery occlusion during intracranial aneurysm surgery. The proposed technique works offline, estimating the SCT before the surgery; however, the authors suggested that an online version would provide a more accurate and precise SCT during the surgery.

A summary of the above-presented studies is comprised in Table 3.

In addition to neurological condition-specific studies, interest has also been drawn to integrating AI algorithms into neurosurgical audits. Brzezicki et al. [39] used the Frideswide algorithm to analyze the same dataset (clinical notes of 45 medical outliers on a neurosurgical ward) as 46 human students. The AI-based algorithm produced considerably more recommendations in a shorter time, the audits being more factually accurate and logically consistent. Thus, this method may help improve the safety, efficiency, and quality of care, implying only a small part of the resources would be required to conduct it through human processes.

### 2.3. Oncology

Cancer comprises a group of more than a hundred types of diseases characterized by abnormal cell growth in different body parts and requires prompt and adequate treatment to prevent serious health issues and increase patients’ survival rates [40,41,42,43]. As cancer poses tremendous burdens on patients and healthcare systems worldwide, there is no surprise that AI has started being investigated in relation to oncology. AI may assist with collecting and evaluating data, diagnose the information on the basis of health, match it with prior information and expertise, and choose adequate diagnostic treatment plans. Thus, it has been studied for improving the diagnosis and management of many forms of cancer, including breast, lung, thyroid, oral, gastric, colorectal, liver, and skin cancers [44].

For instance, Rocca et al. [45] used AI models for improving the radiological diagnosis of colorectal cancer liver metastases (CRCLM). With a precision of 100%, Formal Methods (FM) can help medical professionals predict the presence of liver metastasis still undetectable when using the standard protocols. Thus, the authors concluded that FM could effectively detect CRCLM, even in very heterogeneous and small clinical samples.

Alternatively, Lu and colleagues [46] employed the Faster RCNN model to create a recognition framework for colorectal cancer tumor sprouting. The model can automatically identify the budding areas from pathological sections and count their numbers in a short time, with high accuracy. Therefore, this method can improve the diagnostic efficacy while also reducing the burden of pathologists in reading the sections.

Lee et al. [47] investigated the use of AI in analyzing pancreatic cancer recurrence after surgery and its determinants. Employing the random forest algorithm, the authors concluded that the major predictors of disease-free survival were tumor size, tumor grade, TNM stage, T stage, and lymphovascular invasion. Thus, this technology represents a promising decision support system for treating patients undergoing surgery for pancreatic cancer; yet, further studies are required to demonstrate its benefits in clinical practice.

A different study, conducted by Pantanowitz et al. [48], focused on aiding in a prostate cancer diagnosis from H&E-stained slides of prostate core needle biopsies. Their computer-assisted diagnostic tool could accurately detect, grade, and evaluate clinically relevant features in digitized slides. These encouraging results suggest that the developed technique could be a useful asset in automating the screening of prostate biopsies for a primary diagnosis, assessing signed-out cases for quality control purposes, and standardizing reporting.

An interesting AI approach for cancer care was proposed by Faron et al. [49]. The authors used an automated DL-based body composition analysis pipeline to predict the outcome in patients with melanoma receiving immune checkpoint inhibitor therapy. The model identified a lowered skeletal muscle mass as an independent predictor of mortality, the patients with such characteristics displaying increased mortality rates up to three years after starting the treatment.

An innovative ML-based strategy for diagnosing laryngeal cancer was reported by Kim et al. [50]. The researchers investigated if an automated voice signal analysis could help to discriminate between patients with laryngeal cancer and healthy individuals. Promising results were obtained, as the deployed method demonstrated a greater performance than trained laryngologists in identifying the diseased.

Recent research was also directed at developing a reliable tool for estimating radiation doses before any planning of head and neck radiation therapy. In this respect, Chan et al. [51] created a machine learning-based clinical decision support system that could predict whether mandible subsites would receive a mean dose higher than 50 Gy. Obtaining promising results, the authors concluded that the implementation of such a dose prediction system would allow for more precise estimations of radiation side effects in specific at-risk organs.

One more study that focused on cancer therapy rather than diagnosis/monitoring was proposed by Houy and Le Grand [52]. The authors used AI technology for computing optimal personalized protocols for temozolomide administration in a heterogeneous population of patients. Every day, the protocol was updated with the feedback from the patients’ reactions to drug administration, resulting in very different personalized protocols between the tested group and the standard maximum tolerated dose protocol. This treatment customization was reflected in a reduced tumor size, on average and patient-wise, while avoiding severe toxicity.

To summarize the discussion on the oncological applications of AI, Table 4 was created.

### 2.4. Hematology

AI approaches have also gathered interest in benign and malign hematology settings, being researched for applications in the diagnosis and prognosis of various forms of leukemia, lymphoma, anemias, and genetic blood disorders [53,54].

Carreras et al. [55] recently employed an algorithm using multilayer perceptron ANN to highlight new markers and predict the overall survival of patients with mantle cell lymphoma (MCL) (a subtype of mature B-cell non-Hodgkin’s lymphoma). The AI tool identified five genes associated with poor survival (i.e., KIF18A, YBX3, PEMT, GCNA, and POGLUT3) and five genes linked with favorable survival (i.e., SELENOP, AMOTL2, IGFBP7, KCTD12, and ADGRG2). By the further use of several ML-based algorithms, the authors obtained high accuracy predictions of the overall survival of MCL patients.

In another recent study, El Hussein et al. [56] investigated the use of a novel AI-based heatmap technique for the objective assessment of proliferation centers in chronic lymphocytic leukemia (CLL). The integrative analysis of the cell nuclear size and mean nuclear intensity model demonstrated a high accuracy in separating the three progression stages, displaying robust diagnosis predictive values.

Alternatively, Boldú et al. [57] used peripheral blood cell images to predict an acute leukemia diagnosis. The authors used a DL-based approach, configuring a system with two sequentially working CNN modules: the first one for recognizing abnormal promyelocytes among other mononuclear blood cell images, and the second module for distinguishing whether the blasts were of myeloid or lymphoid lineage. The as-designed model represents a promising asset for clinical pathologists, helping them diagnose acute leukemia during a blood smear review. In comparison, Didi and colleagues [58] trained and compared ML and DL predictive models in order to predict the best treatment for newly diagnosed acute myeloid leukemia. The AI algorithms outperformed the classical statistical analysis or naïve predictors, also predicting the overall survival with high accuracy.

A study proposed by AlAgha et al. [59] focused on overcoming the challenges in a thalassemia diagnosis. Using simple laboratory test results, the authors involved a hybrid data mining approach to differentiate between healthy individuals and persons carrying beta-thalassemia. In addition to the promising performance of the proposed model, this method also helped to reduce the diagnosis cost and time as, without AI tools, additional tests would have been needed to correctly identify the diseased persons.

A different study, conducted by Memmolo et al. [60], aimed to find better solutions for the early differential diagnosis of anemia. The authors used label-free holographic microscopy coupled with a hierarchical ML decider, obtaining enough accuracy for discerning between different hereditary anemia classes with minimal morphological differences. In addition, the method only requires a fraction of a blood drop, reducing the necessary volume of blood drawn for a correct diagnosis. Moreover, this study opened the door for point-of-care blood testing and telemedicine with lab-on-chip platforms.

Given the significant interindividual variabilities of presentation and the clinical course among patients with sickle cell anemia, Dutra et al. [61] proposed an AI method for better understanding this disease. By using a cluster analysis, the authors identified five clusters differentiated by unconjugated bilirubin, reticulocytes, lactate dehydrogenase, leukocytes, lymphocytes, and monocytes. Furthermore, it was investigated if this grouping could be correlated with clinical manifestations, concluding that the clusters exhibited different degrees of inflammation, hemolysis, and liver abnormalities.

Research has also been conducted towards improving anemia management in hemodialysis patients. Barbieri et al. [62] developed an AI decision support system called the “Anemia Control Model” (ACM) that can recommend suitable erythropoietic-stimulating agent doses according to patient profiles. ACM showed promising results, as it helped improve anemia outcomes in the target population while minimizing their medication use and reducing the cost of treatment.

As cytomorphology represents the gold standard for assessing peripheral blood and bone marrow samples in hematological neoplasms, interesting investigations have also been performed for enhancing this method through AI tools. In this respect, Haferlach et al. [63] conducted a prospective, blinded clinical study (NCT04466059) in which they compared a blood smears analysis done by an ML-based model vs. routine diagnostics, obtaining a correlation of 95% for the pathogenic cases. Alternatively, Osman et al. [64] tackled the benefits of a CNN for separating monocytes from their precursor cells (i.e., promonocytes and monoblasts). The authors concluded that the AI-based method could reach an accuracy comparable to the human reviewers, suggesting that CNN models could be used for this task and further improved with a larger study population.

The studies discussed above are outlined in Table 5.

### 2.5. Nephrology

The advancement of digitalization and widespread availability of EHR have been reflected in finding better solutions of care in nephrology settings. AI influence has been specially investigated for the early detection and prediction of acute kidney injury (AKI) in an effort to help clinicians intervene during what may be a crucial stage for preventing permanent kidney injury [65,66].

For example, Tomašev et al. [67] developed a DL approach involving an RNN for the continuous risk prediction of future deterioration in patients with AKI. This method may ensure the identification of patients at risk within a time window that would enable early treatment and, consequently, improve the outcomes.

In contrast, other scientists have employed several ML algorithms for better tackling AKI recognition and prediction. Specifically, Mohamadlou et al. [65] used XGBoost to train an AKI prediction tool on retrospective data. The promising results obtained by the authors encouraged them to state that this method may provide important prognostic capabilities for determining which patients are likely to suffer AKI, but, being a retrospective study, no conclusions could be drawn about the impact on patient outcomes in a clinical setting. Alternatively, Adhikari et al. [66] proposed an algorithm, called “Intraoperative Data Embedded Analytics” (IDEA), to readjust the preoperative risk by the use of a physiological time series and other data collected during surgery. The dynamic incorporation of intraoperative data resulted in improving the postoperative AKI predictions with high sensitivity and specificity. ML algorithms also proved useful in recognizing AKI in burn patients, as demonstrated through studies conducted by Tran et al. [68] and Lin et al. [69].

Another category of nephrological conditions that gathered attention for AI utilization is represented by chronic kidney disease (CKD) in its various forms, including immunoglobulin A nephropathy (IgAN), diabetic kidney diseases (DKD), and autosomal dominant polycystic kidney disease (ADPKD) [3].

For instance, Chen et al. [70] used the XGBoost algorithm coupled with a survival analysis to stratify the risk for kidney disease progression in the setting of IgAN. By using routinely available characteristics, such as urine protein excretion, global sclerosis, and tubular atrophy/interstitial fibrosis, the model could accurately predict the outcome. In comparison, Schena et al. [71] recently developed an ANN prediction model for end-stage kidney disease in patients with IgAN. Being a retrospective study, the authors could compare the predicted and observed outcomes, reporting similar results over a 25-year follow-up period.

A distinct study conducted by Makino and colleagues [72] focused on better understanding DKD. The authors developed a predictive model for DKD aggravation by the use of AI, processing natural language and longitudinal data with big data machine learning. The proposed model may assist in a more effective and accurate intervention towards reducing hemodialysis.

AI can also be employed for automatically determining an estimated glomerular filtration rate (eGFR) and CKD status. In this respect, Kuo et al. [73] used a CNN, called ResNet, to predict kidney function based on ultrasound images, obtaining good performance for the model. Alternatively, Li et al. [74] exploited an ANN integrated with more independent variables for developing an accurate GFR estimation model for the Chinese population. The authors suggested that such a model could fully utilize the predictive ability of additional auxiliary variables but requires further validation in more diverse cohort data.

The potential of AI has also been investigated for predicting dry weight in hemodialysis patients [75], predicting the calciphylaxis risk and understanding the disease mechanism [76], analyzing histopathological images towards improving the diagnostic accuracy of clinicians [77,78], enhancing risk stratification for kidney transplant recipients [79], and early detecting of acute renal transplant rejection [80].

The studies discussed above are shown in Table 6.

### 2.6. Gastroenterology and Hepatology

The fields of gastroenterology and hepatology have also gathered renewed interest in using AI for improving the prediction, diagnosis, treatment, and prognosis of various conditions. For instance, Cao et al. [81] comparatively examined DBN, MLR, and CNN for predicting a long-term postoperative health-related quality of life after bariatric surgery. Among the tested models, DBN showed the best performance; yet, the authors considered that a hybrid network is worth investigating in the future.

Significant attention has been drawn to creating better assessment methods for celiac disease, as this condition may be associated with severe reactions (e.g., pancreatic exocrine dysfunction, microscopic colitis, and enteropathy-associated lymphoma), despite gluten-free diet adherence [10]. In this respect, Caetano dos Santos et al. [82] applied an ML algorithm that enabled a quick and precise endomysial autoantibody (EmA) test analysis for diagnosing celiac disease. In contrast, Syed and colleagues [83] combined a CNN with a deconvolutional network in a histopathological analysis model for identifying and differentiating between duodenal biopsies from children with environmental enteropathy and celiac disease. Alternatively, Choung et al. [84] aimed to discover celiac disease biomarkers derived from neoepitopes of deamidated gliadin peptides (DGP) and tTG fragments. Given the promising results obtained using a SVM, the authors concluded that this biomarker assay can be employed to detect and monitor patients with celiac disease.

Interesting recent developments have also been envisaged for procedures like endoscopy and colonoscopy. Yang et al. [85] used AI image recognition to obtain digestive endoscopy images, judge the disease type, and decide the treatment plan. Their 5G DL edge algorithm exhibited a high accuracy and speed, being considered promising for assisting in a medical diagnosis. On a different note, Zhou et al. [86] aimed to improve the bowel preparation needed for an effective colonoscopy through AI tools. The authors developed an objective and stable DCNN-based system, called ENDOANGEL, whose performance recommends it for application in clinical settings.

Numerous studies have investigated the application of AI tools for detecting, enhancing decision support, and quantifying the treatment response in patients with liver diseases. For instance, Mostafa et al. [87] proposed the use of ML classification methods for the prediction of liver disease in blood donors. Obtaining promising performance metrics, the tested methods (i.e., ANN, random forest, and SVM) could assist healthcare workers in distinguishing between healthy and diseased individuals. A different study conducted by Taylor-Weiner et al. [88] applied machine learning to characterize the disease severity and heterogeneity and quantify the treatment response in nonalcoholic steatohepatitis (NASH). The use of a DCNN led to encouraging results, paving the way for further advances in understanding the disease heterogeneity in NASH, risk stratifying the affected patients, and facilitating the development of therapies. Alternatively, Roy and colleagues [89] introduced a deep learning-based region–boundary integrated network for precise steatosis quantification that can enhance liver disease decision support using whole slide liver histopathology images. In another study, Gawrieh et al. [90] evaluated the role of AI in the detection and quantification of hepatic fibrosis in nonalcoholic fatty liver disease (NAFLD) biopsies. The automated quantification of a collagen proportionate area (CPA) was in good agreement with the pathologist score of the fibrosis stage, demonstrating reliability in accomplishing the set task.

Important works have also been reported in the subfield of liver transplantation, as the many decisions that need to be accomplished for such a procedure could benefit from the integration of AI [9]. Pérez-Sanz et al. [91] recently developed a computer vision-based application for the quantification of macrovesicular steatosis in histopathological liver section slides in pretransplant liver biopsies. The use of a Sudan stain was reflected in a reliable contrast and the facilitation of fast and accurate quantification through the tested ML algorithms. A similar approach was also proposed by Narayan et al. [92], who used a computer vision AI platform to score donor liver steatosis and compared its capability for predicting early allograft dysfunction. This method resulted in slightly better calibration than pathologist steatosis, paving the way for more accurate and reliable predictions of the post-transplantation outcomes.

The discussion on the clinical applications of AI in gastroenterology and hepatology is summarized in Table 7.

### 2.7. Orthopedics and Rheumatology

The use of AI has also reached the interconnected fields of orthopedics and rheumatology, being studied for a variety of applications. For example, Diaz-Rodriguez et al. [94] focused on developing a novel intraarticular (IA) injection for osteoarthritis management. The authors proposed the combination of poloxamers with hyaluronic acid in producing suitable beta-lapachone-loaded IA formulations. With the use of AI, an optimized formulation was developed based on the experimental results of a broad range of hydrogels. According to an ex vivo evaluation, the as-designed formulation exhibited excellent rheological properties and significantly decreased the secretion of degradative and proinflammatory molecules, being a promising candidate for osteoarthritis treatment.

A recent study proposed by Bayramoglu et al. [95] tackled the potential of analyzing patellar bone texture to predict patellofemoral osteoarthritis. Using knee lateral view radiographs, a ML model, and DCNNs, the authors obtained promising results, demonstrating that the analyzed texture features contained useful information of the patellar bone structure and could be used as additional imaging biomarkers in osteoarthritis diagnostics.

Another possible application of AI is the detection and characterization of a meniscus tear based on MRI examinations of the knee. In this respect, Roblot et al. [96] proposed the use of a fast region CNN for a meniscus tears diagnosis, demonstrating its accuracy in detecting the positions of the two meniscal horns, the presence of a meniscal tear, and the orientation of the tear. Similarly, Couteaux et al. [97] trained a mask region-based CNN with MR images to explicitly localize normal and torn menisci and classify the orientation of the tear. The model had a satisfactory performance; yet, the authors concluded that further extension of the database or the inclusion of 3D data could improve the results, especially for nontypical cases of extensively damaged menisci or multiple tears.

Rouzrokh et al. [98] studied the potential of a CNN model for assessing the risk of dislocation following total hip arthroplasty. Based on postoperative anteroposterior pelvis radiographs, the model could be coupled with clinical risk factor information for the rapid and accurate assessment of the risk of dislocation.

Interesting studies have also reported the use of genetic backgrounds for training AI tools, as genetic or epigenomic datasets can be employed in developing new biomarkers and finding new disease patterns and abnormalities [7]. In this context, Patrick et al. [99] utilized data from patients with genotyped psoriatic arthritis (PsA) and cutaneous-only psoriasis (PsC) to train ML algorithms for identifying differences in the genetic architecture between the two groups and assess the PsA risk before the appearance of symptoms. The authors demonstrated that the combination of statistical and ML techniques accurately identified the underlying genetic differences between the psoriasis subtypes, being of potential use in an individualized subtype risk assessment. Alternatively, another research group focused on differentiating rheumatoid arthritis (RA) from osteoarthritis (OA). Specifically, by means of ML algorithms, Long and colleagues [100] found a 16-gene signature, including TMOD1, POP7, SGCA, KLRD1, ALOX5, RAB22A, ANK3, PTPN3, GZMK, CLU, GZMB, FBXL7, TNFRSF4, IL32, MXRA7, and CD8A, that could effectively differentiate RA from OA. Given the model’s good performance, the authors concluded that the proposed genetic signature coupled with complex classification methods holds promise for improving the diagnosis and management of RA patients.

Studies with at least one of the accuracy, precision, sensitivity, or specificity performance metrics available are summarized in Table 8.

### 2.8. Other Applications

Unlike AI in medicine, which uses autonomously functioning algorithms for analyzing patient data towards improving patient outcomes, AI can also be employed in surgery with the involvement of movement. Through the latest developments in DL and DCNNs, AI enables object detection and tracking, making surgical resection easier and safer [101,102]. AI may also be engaged in surgical education for the assessment of surgical competencies, yet further evidence is required concerning its implementation and applicability [103].

The use of AI technologies also paves the way for creating patient-specific devices that can meet the exact requirements of each individual. Interest has been raised in constructing customized devices with distinct designs compared to the commercially available ones that match the anatomic particularities, physiological conditions, and pathological status of patients [104,105]. Specifically, advances have been reported in developing a broad range of personalized devices, including bioprosthetic heart valves [106], cardiovascular stents [107], tissue-engineered vascular grafts [108], prostheses for tumor reconstruction [109], cranial implants [110,111,112], and dental implants [113].

Another potential use of AI consists of contributing to clinical trial designs and execution towards enhancing the participation and diversity within the trial populations. Specifically, the ingenious use of data in the EHR, medical literature, and trial databases can improve patient–trial matching and recruitment, subsequently increasing trial success rates [114,115].

**Figure 3:**
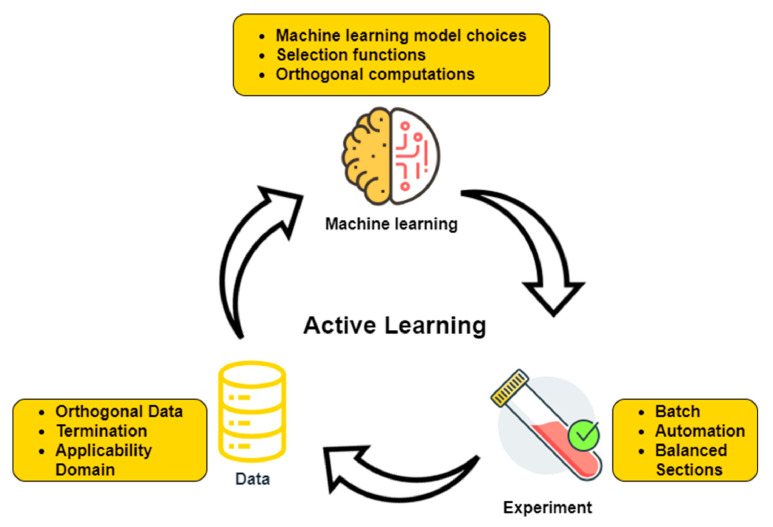
ML in drug discovery. Reproduced from [14], Elsevier B.V. 2021.

**Figure 4:**
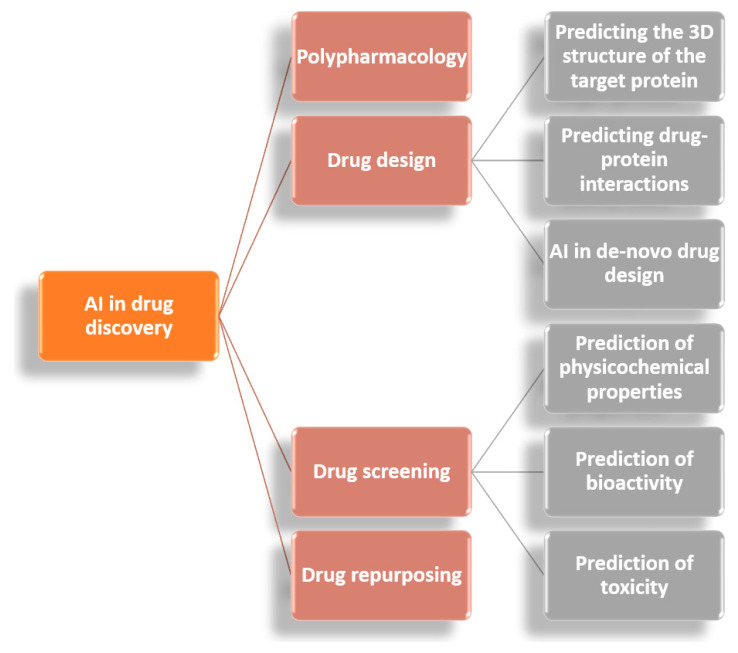
Applications of AI in drug discovery. Adapted from [14].

Promising perspectives also arise from the involvement of AI in the pharmaceutical industry [116,117,118,119]. Various techniques can assess the severity of a disease and predict whether a certain treatment will be effective for an individual patient even before its administration. Moreover, AI can be employed in developing or extrapolating new applications of instruments or chemicals. Particularly, through active learning, ML-based tools can overcome concerning issues in drug design due to their ability to adapt surplus amounts of data available for generating meaningful insights [14] (Figure 3). In addition, introducing AI in studies of polypharmacology, drug design, drug screening, and drug repurposing (Figure 4) can significantly improve the efficiency and reduce the necessary time for generating treatments for various diseases, including new outbreaks like COVID-19 [120].

## 3. Challenges in AI Clinical Integration

**Figure 5:**
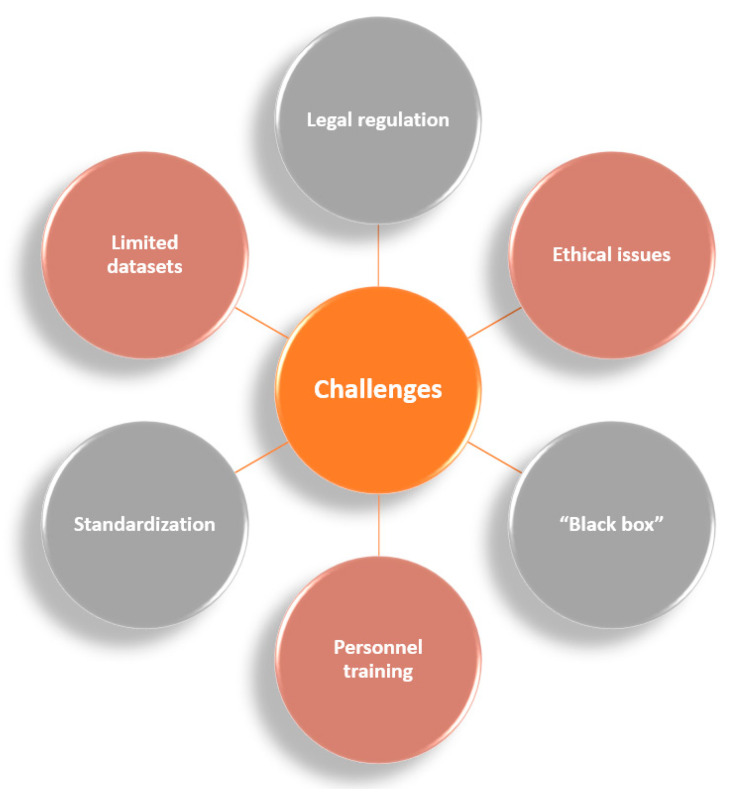
The main challenges in AI clinical integration.

Despite the recent advances in AI technology and the benefits it may bring to clinical care, there are many challenges that impede its translation into practice (Figure 5). One issue is related to the legal regulation of the conditions and features of development, functioning, applicability, integration into other systems, and control over the utilization of end-to-end digital AI technology. As there is no common legal framework yet, this challenge is overcome in each country by considering the particularities of the local legal system [5].

In addition to the rule of law, nonlegal instruments also set important guidelines in medical activities. Hence, AI technology must also be seen from the perspective of the psychological, ethical, and moral aspects of treating patients [5,121].

Another important problem faced by AI tools is skepticism, especially given by the lack of understanding of the methodology of the algorithms [14]. Found in the literature as the “black box” phenomenon, this challenge can be defined as the “human inability in explaining the precise steps leading to the AI tools’ predictions” [11]. Hence, clinicians may preferentially opt for highly transparent models in which the risk factors are handled in a comprehensible way from a pathophysiological point of view. Examples of “black box” techniques are neural networks, random forests, and gradient boosting models, while, at the opposite end, “white box” algorithms can be found, such as logistic regression and decision trees. Nonetheless, there is a tradeoff between the accuracy and interpretability of these methods, causing an ongoing debate of choosing the best options for clinical applications [4].

Although there is a positive attitude towards engaging AI technology in clinical practice, it has been reported that there is a lack of training in students and medical doctors who are supposed to work with these innovative methods. This aspect represents an important drawback, as running AI procedures by inexperienced users may lead to biased, subjective outcomes. This problem can be solved by expanding and improving medical school training in AI through familiarizing healthcare workers and taking full advantage of these emerging technologies without disregarding ethical considerations [121].

Special consideration must be given to the standardization of the metrics used in AI-based studies, as researchers present their findings in quite a heterogeneous manner. In more detail, numerous performance metrics, including accuracy, sensitivity, specificity, precision, F1 score, the area under the receiver operating curve, and more, are alternatively used for demonstrating the quality of a model, yet they are difficult to compare and correlate. Additionally, the outstanding results of exclusive in silico studies may not be reflected with the same success in clinical practice [10,11].

In comparison to business and industry, the medical sector also faces technological limitations in acquiring and analyzing data. As the extent of the resources required to store and analyze data can be prohibitive, it imposes additional limitations on translating AI into clinical investigations and practices. Moreover, the poor organization and management of big data in healthcare may lead to the production of inaccurate models in which erroneous data is inappropriately included [1,122].

Furthermore, ML algorithms are dependent on a predefined set of data to learn from, being restricted by the information that a dataset can provide. When there is a disproportionate number of features in comparison to the amount of data in the training set, the ML model may result in overfitting, compromising the reliability of future predictions and leading to poor generalizability of the findings. Thus, for an AI system to be effectively used in medicine, sufficient data must be provided in the training stage [10,123,124]. Generally, 70% of the available dataset is allocated for training, while the remaining 30% is used for validation. Concerning the data amount, the rule is “the more data, the better”; however, a clear minimum acceptable dataset size has not been indicated. Nonetheless, learning curves (the model performance as a function of the training sample size) may provide an indication of the sample size required for effective training of the model [125,126]. In addition, an algorithm applied in one environment will not automatically be suitable in another environment, requiring careful development, testing, and evaluation in each new context before implementing AI systems for patient care [127]. Intense debate has also arisen from the fact that AI studies tend to compare algorithm performances to clinicians when, instead, realistic applications would involve a combination of human and artificial intelligence [128]. This has raised concerns on whether human clinicians will become redundant with the advancements of AI technology or lose the skills they do not regularly use. Moreover, automation bias means that humans tend to agree with AI decisions, even when they are incorrect. However, as machines cannot be held responsible for their decisions, the legal liability will still be on the shoulders of physicians [10,127,128].

## 4. Conclusions

To summarize, artificial intelligence holds great promise for revolutionizing clinical care. By the ingenious use of big data in healthcare, ML algorithms, and neural networks, better options can be envisaged for the triage, diagnosis, prognosis, monitoring, and treatment of various challenging diseases. Numerous studies have tackled the potential use of AI in medical fields, such as cardiology, neurology, oncology, hematology, nephrology, gastroenterology, hepatology, orthopedics, and rheumatology, and in auxiliary areas, including drug design and the fabrication of patient-specific medical devices.

Nonetheless, several limitations have impeded the translation of AI developments into clinical practices. Thus, prompt solutions and clarifications are required before integrating these emerging technologies into medical protocols. Moreover, the idea that AI should be viewed as a complementary tool to the expertise of clinicians and not as a replacement alternative must be reinforced.

In conclusion, AI is a source of tremendous opportunity in clinical care, deserving considerable attention from the scientific community in order to fully understanding its benefits and develop novel tools. Through the proper exploitation of AI advantages, personalized medicine may soon become a worldwide reality.

## Figures and Tables

**Table 1:** Important AI-related terms and definitions.

Term	Description	References
Machine learning (ML)	Process by which an algorithm encodes statistical regularities from a database of examples into parameter weights for future predictions	[11]
Deep learning (DL)	Multilayered complex ML platform comprised of numerous computational layers able to make accurate predictions	[6]
Supervised learning	Training an ML algorithm using previously labeled training examples, consisting of inputs and desired outputs provided by an expert	[7,11]
Unsupervised learning	When an ML algorithm discovers hidden patterns or data groupings without the need for human intervention	[11]
Reinforcement learning	Learning strategies towards acting optimally in certain situations with respect to a given criterion; such an algorithm obtains feedback on its performance by comparison with this criterion through reward values during training	[7]
Model	A trained ML algorithm that can make predictions from unseen data	[11]
Training	Feeding an ML algorithm with examples from a training dataset towards deriving useful parameters for future predictions	[11]
Features	Components of a dataset describing the characteristics of the studied observations	[1]
Decision tree	Nonparametric supervised learning method visualized as a graph representing the choices and their outcomes in the form of a tree; each tree consists of nodes (attributes in the group to be classified) and branches (values that a node can take)	[12,13]
Random forest	Ensemble classification technique that uses “parallel ensembling”, fitting several decision tree classifiers in parallel on dataset subsamples	[13]
Naïve Bayes (NB)	Classification technique assuming independence among predictors (i.e., assumes that the presence of a feature in the class is unrelated to the presence of any other feature)	[12]
Logistic regression	Algorithm using a logistic function to estimate probabilities that can overfit high-dimensional datasets, being suitable for datasets that can be linearly separated	[13]
K-nearest neighbors (KNN)	“Instance-based learning” or a non-generalizing learning algorithm that does not focus on constructing a general internal model but, rather, stores all instances corresponding to the training data in an *n*-dimensional space and classifies new data points based on similarity measures	[13]
Support vector machine (SVM)	Supervised learning model that can efficiently perform linear and nonlinear classifications, implicitly mapping their inputs into high-dimensional feature spaces	[12]
Boosting	Family of algorithms converting weak learners (i.e., classifiers) to strong learners (i.e., classifiers that are arbitrarily well-correlated with the true classification) towards decreasing the bias and variance	[12]
Artificial neural network (ANN)	An ML technique that processes information in an architecture comprising many layers (“neurons”), each inter-neuronal connection extracting the desired parameters incrementally from the training data	[6,11]
Deep neural network (DNN)	A DL architecture with multiple layers between the input and output layers	[11]
Convolutional neural network (CNN)	A class of DNN displaying connectivity patterns similar to the connectivity patterns and image processing in the visual cortex	[11]

**Table 2:** Summary of the recent AI studies in cardiology.

Task/Objective	AI Tool(s)	Data/Validation	Performance	Ref.
Prediction of incident essential hypertension within the following year	XGBoost	EHR from Maine Health Information Exchange network: Retrospective—*n* = 823,627, calendar year 2013 Prospective—*n* = 680,810, calendar year 2014	Predictive accuracy: Retrospective—91.7% Prospective—87.0%	[18]
Detection of AF using smartwatch data	DNN	9750 participants from Health eHeart Study and 51 patients undergoing cardioversion at the University of California, San Francisco (Enrollment period: February 2016—March 2017)	External validation: Sensitivity—98.0% Specificity—90.2% Exploratory analysis based on self-report of persistent AF in ambulatory patients: Sensitivity—67.7% Specificity—67.6%	[19]
Detection of VF	DL based on 1D-CNN and LSTM network	Public repositories of arrhythmia (*n* = 87,919) OHCA data recorded by monitor defibrillators during treatment in Akershus (Norway), Stockholm (Sweden), and London (UK) between 2002 and 2004 (*n* = 10,857)	For 4-s ECG segments Public data: Balanced accuracy—99.3% Sensitivity—99.7% Specificity—98.9% OHCA data: Balanced accuracy—98.0% Sensitivity—99.2% Specificity—96.7%	[20]
Classification of AS based on cardio-mechanical signals from noninvasive wearable inertial sensors	Elastic Net (for reducing the features generated by CWT) Several ML algorithms 2D-CNN	21 AS patients and 13 non-AS subjects	After the reduction of features by 95.47%, the following accuracies were reported: Decision tree: 87% Random forest: 96% Simple neural network: 91% XGBoost: 95% 2D-CNN: 91% Custom-constructed classifier: 89%	[21]
Feasibility and potential clinical role of FFR_CT_ in patients presenting to the emergency department with acute chest pain who underwent CPCT	ML-based software	56 patients with acute chest pain who underwent CPCT and who had at least a mild (≥25% diameter) coronary artery stenosis	Feasibility—68%	[22]
SCA detection on ECG signal	CNN (for feature extraction) Boosting classifier	57 records from Creighton University Ventricular Tachyarrhythmia Database and MIT-BIH Malignant Ventricular Arrhythmia Database, where each record corresponds to an individual patient The data were divided into 70% of training and 30% of evaluation sets corresponding to 40 and 17 records, respectively	Validated accuracy—99.26% Sensitivity—97.07% Specificity—99.44%	[23]
Clinical measurement of RV and LV volume and function across cardiac MR images obtained for various clinical indications and pathologies	DL algorithm	First 200 noncongenitally clinical cardiac MRI examinations from June 2015 to June 2017 for which volumetry was available	Correlation between automated measurements and manual measurements RV: End systolic volume: r = 0.93 (*p* < 0.001) End diastolic volume: r = 0.92 (*p* < 0.001) Ejection fraction: r = 0.73 (*p* < 0.001) LV: End systolic volume: r = 0.99 (*p* < 0.001) End diastolic volume: r = 0.97 (*p* < 0.001) Ejection fraction: r = 0.94 (*p* < 0.001)	[24]
Classification of various arrythmias from single-lead ECGs	DNN	91,232 single-lead ECGs from 53,549 patients who used a single-lead ambulatory ECG monitoring device	Specificity | Sensitivity for: Atrial fibrillation and flutter—94.1% | 86.1% AVB—98.1% | 85.8% Bigeminy—99.6% | 92.1% EAR—99.3% | 44.5% IVR—99.1% | 86.7% Junctional rhythm—98.4% | 72.9% Noise—98.3% | 80.3% Sinus rhythm—85.9% | 95.0% SVT—98.3% | 48.7% Ventricular tachycardia—99.6% | 70.2% Wenckebach—98.6% | 65.1%	[25]

1D—one-dimensional; 2D—two-dimensional; AVB—atrioventricular block; CWT—continuous wavelet transform; CNN—convolutional neural network; DL—deep learning; DNN—deep neural network; EAR—ectopic atrial rhythm; IVR—idioventricular rhythm; LSTM—long short-term memory; LV—left ventricle; OHCA—out-of-hospital cardiac arrest; RV—right ventricle; SVT—supraventricular tachycardia.

**Table 3:** Summary of the recent AI studies in neurology.

Task/Objective	AI Tool(s)	Data/Validation	Performance	Ref.
Prediction of ischemic stroke recurrence	Several ML algorithms	Geisinger EHR of 2091 ischemic stroke patients	Recurrence within 1-year prediction using random forest with up-sampling the training dataset: Accuracy—88% Positive predictive value—42% Specificity—96%	[27]
Automated lesion detection	ANN classifier	61 patients with pharmacoresistant epilepsy and histologically proven FCD type II from three different epilepsy centers Normal database with 120 healthy controls Additional 35 healthy test controls and 15 disease test controls with histologically confirmed hippocampal sclerosis	Sensitivity—73.7% Specificity—90.0% (91.4% specificity in healthy test group; 86.7% specificity in disease test group)	[31]
Early prediction of epileptic seizures	DCNN Several DL-based classifiers	Long-term scalp EEG data for 22 pediatric subjects with intractable seizures from Children’s Hospital Boston	Accuracy—99.66% Sensitivity—99.72% Specificity—99.60% False alarm rate—0.004 h^−1^	[33]
Prediction of Alzheimer disease status	DL framework linking an FCN to a traditional MLP	Four distinct datasets: ADNI: 229 normal cognition; 188 Alzheimer’s disease AIBL: 320 normal cognition; 62 Alzheimer’s disease FHS: 73 normal cognition; 29 Alzheimer’s disease NACC: 356 normal cognition; 209 Alzheimer’s disease	ADNI test: Accuracy—96.8 ± 1.4% Sensitivity—95.7 ± 1.4% Specificity—97.7 ± 3.1% AIBL: Accuracy—93.2 ± 3.1% Sensitivity—87.7 ± 3.2% Specificity—94.3 ± 4.2% FHS: Accuracy—79.2 ± 3.9% Sensitivity—74.2 ± 18.5% Specificity—80.8 ± 8.2% NACC: Accuracy—85.2 ± 3.7% Sensitivity—92.4 ± 2.5% Specificity—81.0 ± 6.8%	[35]
Diagnosis of Parkinson disease	CNN	45 patients with Parkinson’s disease, 20 patients with atypical parkinsonian syndromes, 35 healthy controls from the general outpatient clinic and movement disorder services at the Department of Neurology, National Institute of Mental Health and Neurosciences, Bangalore, India	Parkinson’s disease vs. healthy controls: Accuracy—80.0% Sensitivity—86% Specificity—70% Parkinson’s disease vs. atypical parkinsonian syndromes: Accuracy—85.7% Sensitivity—100% Specificity—50%	[36]
Assessment of collateral flow of patients with AIS	CNN	200 patients with AIS who presented at the comprehensive stroke center with stroke-like symptoms between March 2019 and January 2020	Dichotomized classification: Accuracy—85 ± 1% Sensitivity—88 ± 1% Specificity—82 ± 3% Multiclass classification: Accuracy—80 ± 1% Sensitivity—64 ± 1% Specificity—85 ± 1%	[28]
Detection of intracranial IED	Template-matching algorithm CNN	1000 intracranial EEG epochs extracted randomly from 307 subjects with refractory epilepsy enrolled in the Defense Advanced Research Projects Agency (DARPA) Restoring Active Memory (RAM) collaborative agreement	Accuracy for classifying an IED—91% Accuracy for classifying a non-IED—96% Sensitivity—91–100% Specificity—82–97%	[34]
Prediction of the safe clipping time of temporary artery occlusion (TAO) during intracranial aneurysm surgery	ANN	125 patients: 105 patients from a retrospective cohort for training the model and 20 patients from a prospective cohort for validating the model	Accuracy—88%	[38]

ADNI—Alzheimer’s Disease Neuroimaging Initiative; AIBL—Australian Imaging, Biomarker and Lifestyle Flagship Study of Ageing; ANN—artificial neural network; CNN—convolutional neural network; DCNN—deep convolutional neural network; FCN—fully convolutional network; FHS—Framingham Heart Study; IED—interictal epileptiform discharges; ML—machine learning; NACC—National Alzheimer’s Coordinating Center.

**Table 4:** Summary of the recent AI studies in oncology.

Task/Objective	AI Tool(s)	Data/Validation	Performance	Ref.
Prediction of liver metastasis presence when still undetectable using the standard protocols	FM	CT scan data of 30 patients collected between January 2013 and June 2021 at the Pineta Grande Hospital Castel Volturno, Caserta, Italy	Precision rate—100% Global accuracy—93.3% Recall rate—77.8%	[45]
Recognition of colorectal cancer tumor sprouting	Faster RCNN	Retrospectively collected 100 surgical pathological sections of colorectal cancer from January 2019 to October 2019; 1000 images were screened, and the total number of tumor buds was approximately 3226	Precision rate—85.5% Image diagnosis accuracy—89% Sensitivity—94% Specificity—83%	[46]
Detection, grading, and evaluation of clinically relevant findings in digitized slides of prostate core needle biopsies	Multilayered CNN	1,357,480 image patches from 549 H&E-stained slides for training; 2501 H&E-stained slides for internal test; external dataset of 100 consecutive cases (1627 H&E-stained slides)	Correlation between cancer percentages calculated by the algorithm and pathologists: r = 0.882 (*p* < 0.0001) Internal test—Sensitivity | Specificity for: Benign vs. cancer—99.59% | 90.14% External validation—Sensitivity | Specificity for: Benign vs. cancer—98.46% | 97.33% Gleason score 6 or ASAP vs. Gleason score 7–10—85.9% | 90.41% ASAP or Gleason pattern 3 or 4 vs. Gleason pattern 5—85% | 90.84% Cancer without vs. with perineural invasion—86.96% | 90.74%	[48]
Automated voice signals analysis for differentiating subjects with laryngeal cancer from healthy individuals	Several ML algorithms	Preoperative medical records from a single university center from July 2015 to June 2019 of patients who underwent voice assessments at the time of laryngeal cancer diagnosis; normal voice samples acquired from otherwise healthy subjects who underwent voice assessments prior to general anesthesia for surgical procedures involving sites other than the head and neck region	Accuracy | Sensitivity | Specificity of: SVM—70.5% | 78.0% | 62.2% XGBoost—70.5% | 62.0% | 80.0% LightGBM—71.5% | 70.0% | 73.3% ANN—69.4% | 62.0% | 77.7% 1D-CNN—85.2% | 78.0% | 93.3% 2D-CNN (MFCCs)—73.3% | 69.6% | 77.5% 2D-CNN (STFT)—67.1% | 58.6% | 76.6%	[50]
Prediction of radiation doses to subsites of the mandible before planning of radiation therapy for oropharyngeal cancer	ML-based clinical decision support	86 previously delivered RT treatment plans (for the training set) and 20 patients whose cases were chronologically subsequent to the training dataset (for the test dataset)	Positive predictive value—95% Negative predictive value—88% Correlation between the prediction of the AI algorithm vs. the physician: r = 0.72 (*p* < 0.001)	[51]

1D—one-dimensional; 2D—two-dimensional; ANN—artificial neural network; ASAP—atypical small acinar proliferation; CNN—convolutional neural network; FM—Formal Methods; MFCCs—Mel-frequency cepstral coefficients; ML—machine learning; RCNN—region convolutional neural network; STFT—short-time Fourier transform; SVM—support vector machine.

**Table 5:** Summary of the recent AI studies in hematology.

Task/Objective	AI Tool(s)	Data/Validation	Performance	Ref.
Automation and enhancement of PCs delineation	CNN	Manually annotated 25, 28, and 21 regions of interest encompassing small round PCs and confluent/expanded PCs of 10 CLL, 12 aCLL, and 8 RT digitized H&E-stained slides, respectively	Accuracy using data from: Nuclear size—65.8 ± 11.5% Mean nuclear intensity—67.9 ± 9.4% Heat value frequencies (integrating nuclear size and mean nuclear intensity)—81.3 ± 6.3%	[56]
Prediction of overall survival and best treatment for acute myeloid leukemia	Several ML algorithms	3687 consecutive adult AML patients included in the DATAML registry between 2000 and 2019 (3030 receiving IC, 657 receiving AZA)	Overall survival prediction accuracy for: Patients receiving IC, at the 18-month mark—68.5% Patients receiving AZA, at the 9-month mark—62.1% Best treatment prediction accuracy—88.5%	[58]
Prediction of diagnosis of acute leukemia using blood cell images	ALNet (a DL model)	A set of 731 blood smears containing 16,450 single-cell images from 100 healthy controls, 191 patients with viral infections and 148 with acute leukemia	Overall accuracy—94.2% Acute promyelocytic leukemia Sensitivity—100% Specificity—100% Precision—100% Acute myeloid leukemia Sensitivity—100% Specificity—92.3% Precision—93.7% Acute lymphoid leukemia Sensitivity—89% Specificity—100% Precision—100%	[57]
Automatic detection of β-thalassemia carriers	CRISP-DM SMOTE (oversampling technique) Several classifiers	Blood parameters of apparently healthy 45,498 individuals who were referred to the Thalassemia and Hemophilia center, Palestine Avenir Foundation in from 2012 to 2016 to be screened for the premarital tests; 44,360 of the study samples were classified as normal while 1138 were confirmed as β-thalassemia carriers	Sensitivity—98.81% Specificity—99.47%	[59]
Differential screening of hereditary anemias from a fraction of blood drop	Hierarchical ML decider Several classifiers	8 patients with clinical and molecular diagnosis of CDA type I, CDA type II, HS, DHS1, IRIDA, and α-thalassemia and 7 healthy donors; for each donor, up to ten independent digital holograms of RBCs were recorded	Overall accuracy of cubic SVM for: Binary classification—84.3% Differential classification—69.5%	[60]

AML—Acute Myeloid Leukemia; AZA—azacitidine; CDA—congenital dyserythropoietic anemia; CNN—convolutional neural network; CRISP-DM—cross-industry standard process for data mining; DHS—dehydrated hereditary stomatocytosis; HS—hereditary spherocytosis; IC—intensive chemotherapy; IRIDA—iron-refractory iron-deficiency anemia; SVM—support vector machine.

**Table 6:** Summary of the recent AI studies in nephrology.

Task/Objective	AI Tool(s)	Data/Validation	Performance	Ref.
Prediction of future acute kidney injury	DL model	Dataset consisting of all eligible patients during a five-year period across the entire Veterans Affairs healthcare system in the USA (703,782 adult patients across 172 inpatient and 1062 outpatient sites) The test population was a random selection of 10% of these, counting 70,681 individual patients and 252,492 unique admissions	Prediction with a lead time of up to 48 h and a ratio of 2 false alerts for every true alert: 55.8% of all inpatient episodes of acute kidney disease 90.2% of all acute kidney injuries that required subsequent administration of dialysis	[67]
Early detection and prediction of acute kidney injury	XGBoost	Patients whose hospital stays lasted between 5 and 1000 h and who had at least one documented measurement of heart rate, respiratory rate, temperature, serum creatinine (SCr), and Glasgow Coma Scale (GCS) (48,582 patients from BIDMC and 19,737 patients from Stanford Medical Center)	Accuracy | Sensitivity | Specificity of prediction for stage 2 or stage 3 acute kidney injury in the BIDMC dataset: Onset—81% | 81% | 75% 12 h before onset—76% | 77% | 62% 24 h before onset—82% | 83% | 56% 48 h before onset—82% | 83% | 48% 72 h before onset—80% | 82% | 45% Accuracy | Sensitivity | Specificity of prediction for stage 2 or stage 3 acute kidney injury in the Stanford dataset: Onset—78% | 77% | 82% 12 h before onset—75% | 75% | 73% 24 h before onset—79% | 79% | 64% 48 h before onset—84% | 85% | 51% 72 h before onset—79% | 78% | 53%	[65]
Postoperative acute kidney injury prediction	IDEA (ML algorithm)	Retrospective single-center cohort of 2911 adults who underwent surgery at the University of Florida Health between 2000 and 2010	Preoperative model: Accuracy—76% Sensitivity—68% Postoperative stacked model Accuracy—78% Sensitivity—80% Postoperative full model Accuracy—80% Sensitivity—81%	[66]
Mortality prediction for acute kidney injury patients in the intensive care unit	Several ML algorithms	Medical information mart for intensive care (MIMIC) III database from 19,044 patients with acute kidney injury among which 2586 died	With the prediction sensitivity fixed at 85%, the following accuracies were reported: Random forest—72.8% SVM—72.9% ANN—66.6% Customized SAPS II—58.0%	[69]
Prediction of diabetic kidney disease progression	CAE	EHR of 64,059 type II diabetes patients	Accuracy—71%	[72]
Automatic determination of the eGFR and chronic kidney disease status	ResNet (CNN)	4505 kidney ultrasound images labeled using eGFRs derived from serum creatinine concentrations	Accuracy—85.6% Precision—91.3% Correlation between AI and creatinine-based GFR estimations—0.741	[73]
GFR estimation	ANN	1959 chronic kidney disease patients (development dataset: 1075 participants from January 2012 to December 2014; validation dataset: 877 participants from January 2015 to June 2016)	Accuracy—75.8%	[74]
Early detection of acute renal transplant rejection	CNN	Diffusion-weighted MRI dataset of 56 individuals (with associated clinical biomarkers), who had renal transplantation	Accuracy—92.9% Sensitivity—93.3% Specificity—92.3%	[80]
Multiclass segmentation of kidney tissue in sections stained by PAS	CNN	Blouin-fixed, paraffin-embedded needle-core biopsies from 101 patients who underwent a kidney transplantation between 2008 and 2012 in the Radboud University Medical Center, Nijmegen, The Netherlands (Radboudumc); 132 PAS-stained slides from Radboudumc pathology archives	Correlation between glomerular counting performed by pathologists vs. AI—0.94	[77]

ANN—artificial neural network; BIDMC—Beth Israel Deaconess Medical Center; CAE—convolutional autoencoder; CNN—convolutional neural network; DL—deep learning; IDEA—Intraoperative Data Embedded Analytics; ML—machine learning; SVM—support vector machine; XGBoost—extreme gradient boosting.

**Table 7:** Summary of the recent AI studies in gastroenterology and hepatology.

Task/Objective	AI Tool(s)	Data/Validation	Performance	Ref.
Prediction of long-term health-related quality of life and comorbidity after bariatric surgery	DBN MLR	6542 patients registered in the Scandinavian Obesity Surgery Registry between 2008 and 2012 operated on with primary Roux-en-Y gastric bypass	Accuracy | Sensitivity | Specificity of DBN for predicting 5-year comorbidities: Sleep apnea syndrome—91% | 64% | 92% Hypertension—84% | 83% | 83% Type 2 diabetes—90% | 96% | 89% Depression—87% | 51% | 95% Dyslipidemia—90% | 78% | 91% Accuracy | Sensitivity | Specificity of MLR for predicting 5-year comorbidities: Sleep apnea syndrome—73% | 90% | 73% Hypertension—68% | 73% | 67% Type 2 diabetes—69% | 78% | 68% Depression—57% | 66% | 55% Dyslipidemia—68% | 76% | 67%	[81]
Assessment of bowel preparation	ENDOANGEL (DCNN)	5583 clear and unambiguous colonoscopy images retrospectively collected from over 2000 patients (for training dataset) 20 retrospectively and randomly collected colonoscopy videos, independent of the images (for testing dataset)	Accuracy: Human-machine contest with 120 images—93.33% 100 images with bubbles—80.00% 20 colonoscopy videos—89.04%	[86]
Automatic assessment and classification of the EmA test for celiac disease	SVM	2597 high-quality IgA class EmA images collected in 2017–2018 in the celiac disease service laboratory at the Tampere University, Tampere, Finland	Accuracy—96.80% Sensitivity—82.84% Specificity—99.40%	[82]
Identification of immunogenic epitopes of the tTG-DGP complex for use in detection and monitoring patients with celiac disease	SVM	Serum samples from 90 patients with biopsy-proven celiac disease and 79 healthy individuals for the training dataset and 82 patients with newly diagnosed CeD and 217 controls for the validation dataset	Identification of patients with celiac disease: Sensitivity—99% Specificity—100% Identification of patients with mucosa healing status: Sensitivity—84% Specificity—95%	[84]
Detection of pathologic morphological features in diseased vs. healthy duodenal tissue	CNN	3118 segmented images from 121 H&E-stained duodenal biopsy glass slides from 102 patients collected between November 2017 and February 2018	Accuracy—93.4%	[83]
Prediction of liver disease	Several ML algorithms	615 patients (blood donors and non-blood donors with Hepatitis C) data collected from the University of California Irvine Machine Learning Repository	ANN Accuracy—88.89% Precision—94.84% Sensitivity—95.23% Specificity—82.88% Random forest Accuracy—98.14% Precision—99.08% Sensitivity—99.04% Specificity—97.29% SVM Accuracy—96.75% Precision—96.42% Sensitivity—96.19% Specificity –97.29%	[87]
Quantification of steatosis, inflammation, ballooning, and fibrosis in biopsies from patients with NAFLD	ML algorithm	Data from 246 consecutive patients with biopsy-proven NAFLD and followed up in London from January 2010 to December 2016; biopsy specimens from the first 100 patients were used for training, while the other 146 were used for validation	Correlation between manual annotation and software results: Steatosis: r = 0.97 (*p* < 0.001) Inflammation: r = 0.96 (*p* < 0.001) Ballooning: r = 0.94 (*p* < 0.001) Fibrosis: r = 0.92 (*p* = 0.001)	[93]
Detection and quantification of hepatic fibrosis and assessment of its architectural pattern in NAFDL biopsies	Supervised ML models	A set of digital images of trichrome stained slides of 18 unique liver biopsies	Precision of fibrosis patterns: Normal—85.6% Pericellular—76.6% Periportal—72.1% Portal—77% Bridging—84.9% Nodule—89.8%	[90]
Automatic objective quantification of macrovesicular steatosis in histopathological liver section slides stained with Sudan	Several ML and DL algorithms	Eight micrometer-thick sections obtained from 20 donor liver samples	Accuracy | Sensitivity | Specificity of KNN—99.6% | 84.4% | 99.9% SVM- 99.6% | 96.2% | 99.7% Random forest 99.6% | 95.6% | 99.7% NB—99.7% | 91.0% | 99.9% Simple NN—99.7% | 96.3% | 99.8% Keras—99.5% | 97.2% | 99.6%	[91]

ANN—artificial neural network; CNN—convolutional neural network; DBN—discrete Bayesian network; DCNN—deep convolutional neural network; KNN—K-nearest neighbors; ML—machine learning; MLR—multivariable logistic regression; NB—Naïve Bayes; NN—neural network; SVM—support vector machine.

**Table 8:** Summaries of the recent AI studies in orthopedics and rheumatology.

Task/Objective	AI Tool(s)	Data/Validation	Performance	Ref.
Automatic knee meniscus tear detection and orientation classification	RCNN	A total of 1128 images, with an imbalanced number of horizontal posterior tears, vertical posterior tears, horizontal anterior tears, and vertical anterior tears	Accuracy—83% Precision—86%	[97]
Assessment of the risk of hip dislocation based on postoperative anteroposterior pelvis radiographs	YOLO-V3 ResNet18 (CNNs)	Retrospective radiographs of 13,970 primary THAs with 374 dislocations after 5 years of follow-up, accounting for 1490 radiographs from dislocated and 91,094 from non-dislocated THAs	Accuracy—49.55% Sensitivity—89.02% Specificity—48.77%	[98]
Prediction of PsA among psoriasis patients	Several ML algorithms	Data from six cohorts with more than 7000 genotyped PsA and PsC patients	For the top 5% of patients predicted as having PsA: Precision—>90% Specificity—100%	[99]
Differential diagnosis of rheumatoid arthritis and osteoarthritis	Several ML algorithms	Affymetrix and Illumina microarrays on gene expression in rheumatoid arthritis and osteoarthritis healthy control synovial tissues curated from Gene Expression Omnibus	Rheumatoid arthritis: Accuracy—86% Sensitivity—100% Specificity—77% Osteoarthritis: Accuracy—85% Sensitivity—90% Specificity—80%	[100]

CNN—convolutional neural network; ML—machine learning; RCNN—region convolutional neural network; PsA—psoriatic arthritis; PsC—cutaneous-only psoriasis; THA—total hip arthroplasty.

## Data Availability

Not applicable.

## Abbreviations

1D	one-dimensional
2D	two-dimensional
aCLL	accelerated chronic lymphocytic leukemia
ADNI	Alzheimer’s Disease Neuroimaging Initiative
AI	Artificial Intelligence
AIBL	Australian Imaging, Biomarker and Lifestyle Flagship Study of Ageing
AIS	acute ischemic stroke
AF	atrial fibrillation
ANN	artificial neural network
AS	aortic stenosis
ASAP	atypical small acinar proliferation
AVB	atrioventricular block
AZA	azacitidine
BIDMC	Beth Israel Deaconess Medical Center
CAE	convolutional autoencoder
CDA	congenital dyserythropoietic anemia
CLL	chronic lymphocytic leukemia
CNN	convolutional neural network
CPCT	chest-pain computed tomography
CRISP-DM	cross-industry standard process for data mining
CWT	continuous wavelet transform
DBN	discrete Bayesian network
DCNN	deep convolutional neural network
DHS	dehydrated hereditary stomatocytosis
DL	deep learning
DNN	deep neural network
EAR	ectopic atrial rhythm
ECG	electrocardiogram
EEG	electroencephalogram
eGFR	estimated glomerular filtration rate
EHR	electronic health record
EmA	endomysial autoantibody
FCD	focal cortical dysplasia
FCN	fully convolutional network
FFR_CT_	computed tomography-derived fractional flow reserve
FHS	Framingham Heart Study
FM	Formal Methods
GENFI	Genetic Frontotemporal dementia Initiative
GFR	glomerular filtration rate
H&E	hematoxylin and eosin
HS	hereditary spherocytosis
IC	intensive chemotherapy
IDEA	Intraoperative Data Embedded Analytics
IED	interictal epileptiform discharges
IRIDA	iron-refractory iron-deficiency anemia
IVR	idioventricular rhythm
KNN	K-nearest neighbors
LightGBM	light gradient boosted machine
LSTM	long short-term memory
LV	left ventricle
MFCCs	Mel-frequency cepstral coefficients
ML	machine learning
MLR	multivariable logistic regression
MRI	magnetic resonance imaging
NACC	National Alzheimer’s Coordinating Center
NAFLD	nonalcoholic fatty liver disease
NB	Naïve Bayes
NLP	multilayer perceptron
NN	neural network
MR	magnetic resonance
PAS	periodic acid-Schiff
PCs	proliferation centers
PsA	psoriatic arthritis
PsC	cutaneous-only psoriasis
RCNN	region convolutional neural network
RNN	recurrent neural network
RT	Richter transformation
RV	right ventricle
SAPS	simplified acute physiology score
SCA	sudden cardiac arrest
STFT	short-time Fourier transform
SuStaIn	Subtype and Stage Interference
SVM	support vector machine
SVT	supraventricular tachycardia
THA	total hip arthroplasty
TNM	tumor, nodes, and metastases
tTG-DGP	tissue transglutaminase-deamidated gliadin peptides
VF	ventricular fibrillation
XGBoost	extreme gradient boosting
